# Resilience to suicidal behavior in young adults: a cross-sectional study

**DOI:** 10.1038/s41598-022-15468-0

**Published:** 2022-07-06

**Authors:** Jin Han, Iana Wong, Helen Christensen, Philip J. Batterham

**Affiliations:** 1 Black Dog Institute, University of New South Wales, Hospital Road, Randwick, Sydney, NSW 2031 Australia; 2 School of Psychology, University of New South Wales, Sydney, Australia; 3 Centre for Mental Health Research, Research School of Population Health, The Australian National University, Canberra, Australia

**Keywords:** Epidemiology, Risk factors

## Abstract

Despite decades of research on suicide risk factors in young people, there has been no significant improvement in our understanding of this phenomenon. This study adopts a positive deviance approach to identify individuals with suicide resilience and to describe their associated psychological and sociodemographic profiles. Australian young adults aged 18–25 years with suicidal thoughts (N = 557) completed an online survey covering sociodemographic, mental health status, emotion regulatory and suicide-related domains. Latent class analysis was used to identify the individuals with suicide resilience. The predictors of suicide resilience were assessed using logistic regression models. The results suggested that one in ten (n = 55) met the criteria for suicide resilience. Factors that had a significant association with suicide resilience included greater cognitive flexibility, greater self-efficacy in expressing positive affect, reduced use of digital technology and less self-harm and substance use as a response to emotional distress. This study identified the factors that may protect young adults with suicidal thoughts from progressing to suicide attempts. Suicide prevention programs might be optimised by shifting from a deficit-based to a strength-based approach through promoting cognitive flexibility, self-efficacy and reducing maladaptive coping.

## Introduction

Suicide is a complex public health phenomenon that represents a leading cause of death for young adults aged between 18 and 25 years globally and in Australia^1^. Research in this field has mostly focused on detecting the risk factors of suicide with a noticeable scarcity of studies on suicide resilience^2–4^. Shifting our attention to resilience to suicide and its related behavior may help inform innovative suicide prevention strategies^5,6^. Developing strengths-based approaches can be an important new direction in developing interventions, as a focus on risks of suicide has not advanced our understanding of suicide or reduced suicide rates^7^.

Suicide resilience is generally acknowledged as both the ability and the dynamic process of maintaining psychological and physical health functioning under high levels of suicide risks^8,9^. The concept is largely derived from well-documented observations that the majority of high-risk individuals, e.g., those with depression^10^ or suicidal thoughts^11^, do not develop suicidal behavior. Most of the current studies on suicide resilience adopt the buffering hypothesis^8^ to identify the psychological constructs that moderate the relationship between suicide risks and suicidal behavior^12,13^. This approach, however, is often limited because it yields relatively small effect sizes (2–5%) of the indicated interaction effects and inconsistent findings across studies, while a person-centered approach by identifying resilient individuals demonstrates the stability of resilience over time^14^.

Positive deviance (PD) is a person-centered research approach that is promising in disentangling the relationship between suicide risks and suicidal behavior. This approach is gaining prominence in identifying and understanding individuals’ exceptional performance in complex health situations^15–17^. The PD approach is grounded on the premise that in each community there are certain individuals whose uncommon behavior and strategies enable them to find better solutions to the same problems than their peers^18^. It has been effectively implemented as a practical strategy to untangle complex health problems, such as child malnutrition^19^, safe sexual practices^20^, and weight control^21^. To our knowledge, the PD approach has not been implemented in suicide prevention.

In this study, we followed the general practice in PD research to first define the problem^22^. We aim to understand the factors that promote resilience to suicidal behavior amongst young people with suicidal thoughts, as the transition from suicidal thoughts to behavior is increasingly acknowledged as a significant predictor of suicide death in theories^23,24^ and empirical literature^23,24^.

One area that warrants further investigation in the suicidal ideation to action framework is emotion regulation^25^. Emotion regulation, known as the individual process that influences one’s experience and expression of emotions^26^, has been suggested to be closely associated with resilience, as “the experience of adversity is inherently emotional^27^”. The positive association between effective emotion regulation and psychological resilience is well documented in the general population^28^ and among individuals with mental illnesses^29–31^, traumas^32–34^, and physical conditions^35^. Although the relationship between effective emotion regulation and suicide resilience is under-investigated, extensive research has indicated that emotion dysregulation is associated with suicidal thoughts and a history of suicidal attempts after taking into account the symptoms of psychological disorders^36–39^.

Emotion dysregulation is closely related to increased risks of engaging in risky behavior^40^, such as deliberate self-harm^41^ and alcohol use^42^. These associations may be mediated by an increased acquired capability for suicide, a proposed key factor in the transition from suicidal thoughts to attempts^43^. However, deficits in emotion regulation also indicate individuals’ low ability to tolerate distress, as the behavior of emotionally dysregulated individuals is often motivated by their desire to escape from painful experiences^44^. Emotion dysregulation can thus serve to counter or lower acquired capability for suicide^45^. The inconsistent findings may be related to different aspects of emotion regulation, such as the adoption of maladaptive emotion regulation strategies such as self-harm behavior^46^, ability to switch between emotional regulation strategies in adaption to situations (flexibility)^47,48^, or confidence in the ability to cope with different stressors (self-efficacy)^49,50^. Therefore, further investigation is required in the context of youth suicide prevention is required to better understand the roles of these related components of emotion regulation.

Therefore, this study seeks to, for the first time, understand resilience to suicidal behavior among young adults with suicidal thoughts using the positive deviance-informed analytic approach. We hypothesized that young people with suicide resilience would be distinguished by more adaptive emotion regulation strategies, greater coping and cognitive flexibility and greater self-efficacy in emotion regulation, after adjusting for constraints (i.e., risk factors for suicidal attempts). We expected that our results would improve our understanding of protective factors to optimise suicide prevention interventions and facilitate the transition from a deficit-based suicide prevention approach to a strength-based one. Identifying psychological, social and behavioral profiles of young people who demonstrate positive deviance may indicate specific therapeutic targets, thereby guiding the development of more effective treatment and prevention interventions for individuals with suicidal thoughts.

## Method

### Participants and procedure

Australian young adults aged between 18 and 25 years who experienced suicidal thoughts in the past year were recruited from Facebook and Instagram advertisements in February 2021. Other eligibility criteria include being fluent in English, currently living in Australia, having no diagnosis of bipolar disorder or psychosis, and having no suicide attempt in the past 30 days. Informed consent was obtained from all participants. Eligible participants then filled in a Qualtrics online survey, including questions on sociodemographic (e.g., age, sex, socioeconomic status), mental and physical health conditions and status (e.g., suicidal thoughts and behavior, depression, anxiety), emotion regulation and related constructs (e.g., coping flexibility, cognitive flexibility, regulatory emotional self-efficacy), and health service usage (e.g., help seeking intentions). The participants were reimbursed for their participation by a draw to win one of three $30 e-gift vouchers. From the 2392 clicks on the Facebook and the Instagram advertisements, 725 participants completed the eligibility assessment, of which 658 (90.8%) were eligible, and 557 (84.7%) completed the variables of interest for the current study. No significant difference was found between the complete and incomplete responses on age (*t* = 0.227, *df* = 655, *p* = 0.821) and sex (*χ*^2^(1) = 0.32, *p* = 0.571). Survey data were further screened for abnormal responses in terms of survey duration and ranges of scale scorings before analysis, with no anomalous responses identified. The mean age of the included participants (N = 557) was 21.9 years (*SD* = 2.3 years). Eighty-four percent were female, 51.2% self-identified as lesbian, gay, bisexual, trans, and/or intersex, 78.1% living in metropolitan areas, and 41.7% married, de facto, or in a relationship. The research was carried out in accordance with the latest version of the Declaration of Helsinki, and the study received ethics approval from the Human Research Ethics Committee at the University of New South Wales (HC200696).

### Measures

*Sociodemographic variables* include date of birth, sex, sexual orientation, current living and relationship status, the highest level of education completed, self-perceived socioeconomic status^51^, history of diagnosed mental illness and long-term physical health conditions, and current medication.

*Mental and physical wellbeing measures* include subjective mental wellbeing measured by the Short Warwick-Edinburgh Mental Well-Being Scale SWEMWBS;^52^. The scale consists of seven items that assess general mental wellbeing over the past two weeks. Total converted scores range from 7 to 35 with higher scores indicating higher levels of subjective mental well-being. This scale has demonstrated good reliability in the current study (Cronbach's α = 0.82). Physical wellbeing was measured by the EQ-VAS^53^, a visual analogue scale numbered from 0 (the worst health you can imagine) to 100 (the best health you can imagine) over the last two weeks. A higher score represents a perception of better physical health.

The severity of suicidal thoughts was assessed by the Suicidal Ideation Attributes Scale SIDAS;^54^, consisting of five items related to the frequency of suicidal thoughts, controllability, closeness to suicidal attempt, levels of distress and impact on daily functioning in the past month. Item two (controllability) is reverse scored. The total scale ranges from 0 to 50, with higher scores indicating more severe suicidal thoughts. The measure has shown good internal consistency (Cronbach's α = 0.84) in the current study. The history of suicide attempts was assessed by a question on a three-point Likert scale, ranging from “No, never (0)”, “Yes, once (1)” to “Yes, more than once (2)”^55^.

Levels of depression and anxiety over the past two weeks were measured using the Patient Health Questionnaire-9 PHQ-9;^56^ and the Generalized Anxiety Disorder-7 GAD-7;^57^. Higher scores indicate more severe depression and anxiety. Both measures have shown good internal consistency: PHQ-9 (Cronbach's α = 0.86) and GAD-7 (Cronbach's α = 0.88). The positive and negative affect was assessed by the International Positive and Negative Affect Schedule Short-form I-PANAS-SF;^58^. The scale has ten items that assess the frequency of positive affect (active, determined, attentive, inspired, and alert) and negative affect (afraid, nervous, upset, hostile, and ashamed) over the last two weeks on a 5-point Likert scale, ranging from “Never (1)” to “Always (5)”. This scale has shown acceptable consistency (Cronbach's α = 0.72 for positive affect and Cronbach's α = 0.68 for negative affect) in the current study.

*Emotion regulation related measures* include the 16-item Brief Difficulties in Emotion Regulation Scale DERS-16;^59^ evaluating emotion regulation difficulties: emotional clarity, goals, impulsivity, strategies, and non-acceptance. Total score ranges from 16 to 80, with higher scores reflecting greater levels of emotion dysregulation. DERS-16 has shown good internal consistency in the current study (Cronbach's α = 0.91).

The levels of flexibility were measured by the Coping Flexibility Scale CFS;^60^ and the Cognitive Flexibility Scale CFS;^61^ respectively. The Coping Flexibility Scale has 10 items, and items 2 and 7 are reverse coded. The total score ranges from 0 to 30 with higher scores indicating a higher ability of coping flexibility. The Cognitive Flexibility Scale comprises 12 items measuring aspects of cognitive flexibility relevant to effective interactions and communication. Items 2, 3, 5, and 10 items are reverse scored and the total scores of the scale range between 12 and 72. Higher scores indicate stronger cognitive flexibility. Both scales demonstrated good internal consistency (Coping Flexibility Scale: Cronbach's α = 0.77, Cognitive Flexibility Scale: Cronbach's α = 0.75) in the current study.

Self-efficacy in emotion regulation was measured by the Regulatory Emotional Self-efficacy Scale RESE;^62^. The scale is composed of 12 items that assess competence belief in regulating affects due to the occurrence of positive or negative events. This scale assesses self-efficacy in three aspects of emotion regulation: expressing positive emotions, managing despondency-distress, and managing anger-irritation. Each aspect is measured by four items, with total scores for each subscale ranging from 4 to 20. All subscales have shown good internal consistency in this study, with Cronbach's α ranging from 0.71 to 0.80.

Cognitive and behavioral responses to emotional distress were measured by 18 questions assessing the likelihood of participants’ engaging in the listed activities^63,64^ using a four-point Likert scale, ranging from “Highly unlikely (1)” to “Highly likely (4)”. The activities cover six aspects, including digital technology (i.e., watching TV or online videos, browsing social media, playing videogames or computer games), creative arts (i.e., jigsaws, drawing or journaling), self-harm and substance use (i.e., using drug or alcohol, self-harm, thinking about death), exercise (i.e., anerobic exercise, aerobic exercise), self-transcendence (i.e., pray, mindfulness or mediation, challenging negative thoughts, doing randomly kind things to others), and selfcare (i.e., sleeping, taking a bath or long shower, playing with pets, massaging), with average scores of each aspect ranging from one to four. Higher scores indicate greater intentions to engage in the activities.

### Statistical analysis

Latent class analysis (LCA) was used to identify the positive deviant (PD) cases. LCA is a mixture modelling technique that classifies a seemingly heterogeneous sample into a discrete number of subgroups (‘classes’), focusing on the similarities and differences across individuals^65^. Whereas LCA is typically seen as a data-driven approach where the best fitting model is chosen based on statistical consideration, theoretical and practical insights can also be used to optimise the model^66^. In this study, the latent class analysis was performed based on three constructs: subjective mental wellbeing measured by the SWEMWBS, physical wellbeing measured by the EQ-VAS, and the existence of self-reported lifetime suicidal attempts. These constructs were chosen to identify PD individuals who maintained high levels of wellbeing and had not attempted suicide to investigate the resilience factors to suicidal behavior. A 2-class, 3-class, 4-class, and 5-class models were specified via Mplus version 8, including Bayesian Information Criterion (BIC), Adjusted Bayesian Information Criterion (ABIC), significant levels of bootstrapped likelihood ratio test (BLRT), and the percentages of the total sample across class membership. The model was selected based on the significant BLRT, the most robust indicator of class membership, and no classes with < 5% of the total sample^67^.

Subsequent analyses were conducted with IBM SPSS Statistics (version 26). To compare the fitting classes on sociodemographic, mental, and physical health conditions, and emotion regulation-related factors variables, independent sample t-tests were conducted for continuous variables, and chi-square tests were conducted for dichotomous variables. The predictors of PD class membership were first assessed using base models that included each individual predictor. Significant predictors were then included in a multivariate logistic regression model to assess the impact while adjusting for potentially confounding effects. Significance levels were set at *p* < 0.05. All analyses were performed using SPSS Version 26 (SPSS Inc, Chicago, IL, USA).

## Results

### Classification of PD and non-PD cases

Tables 1, 2 displays the fit statistics for the latent class analysis (Table 1) and the description of latent classes (Table 2). This study tested models with 2–5 classes, all of which were supported by BLRT except the 5-class model. Although ABIC and BIC supported a 5-class model, membership of the fifth class in the model was low, comprising 3.1% of the total sample. The 3-class and the 4-class models also included a class of less than 5% of the total sample. Consequently, the 2-class model was retained in this study. Membership for the 2-class model was as follows: Class 1 had 502 individuals (90.1% of the total sample) with 53.4% who reported lifetime suicidal attempts and significantly lower subjective mental (17.5 vs 22.4, *t* = −13.18, *df* = 555, *p* < 0.001) and physical (46.3 vs 79.1, *t* = −18.96, *df* = 100.6, *p* < 0.001) wellbeing than Class 2 (n = 55, 9.9% of the total sample). Participants in Class 2 hereafter are referred to as positive deviant (PD) cases who demonstrated resilience to suicidal behavior and high mental and physical wellbeing.

**Table 1:** Fit statistics and class membership for the identification of PD cases (n = 557).

**Class membership**								
Model	BIC	ABIC	BLRT	1	2	3	4	5
2-class	8592.4	8567.0	< .001	502 (90.1%)	55 (9.9%)			
3-class	8416.8	8378.7	< .001	305 (54.8%)	237 (42.5%)	15 (2.7%)		
4-class	8375.4	8324.6	.0002	302 (54.2%)	208 (37.3%)	29 (5.2%)	18 (3.2%)	
5-class	8345.9	8282.4	.0813	262 (47.0%)	199 (35.7%)	48 (8.6%)	31 (5.6%)	17 (3.1%)

PD: Positive Deviant; BIC: Bayesian Information Criterion; ABIC: Adjusted Bayesian Information Criterion; BLRT: Bootstrapped Likelihood Ratio Test for k versus k–1 classes; class membership is sorted from largest to smallest.

**Table 2:** Concordance of group membership in suicidal attempts and mental and physical wellbeing (n = 557).

2-class	Class 1 (n = 502)		Class 2 (n = 55, PD)			
	*n*	*%*	*n*	*%*	χ2	*p*
Lifetime suicidal attempts	268	53.4%	0	0.0%	56.59	< .001
	Mean (SD)	Range	Mean (SD)	Range	t	p
SWEMWS mental wellbeing	17.5 (2.7)	7.0–30.7	22.4 (2.4)	17.4–29.3	−13.18	< .001
EQ_VAS physical wellbeing	46.3 (20.2)	0.0–95.0	79.1 (10.9)	40.0–100.0	−18.96	< .001

SD: Standardised Deviance; PD: Positive Deviant; SWEMSWS: short Warwick–Edinburgh Mental Well-being Scale; EQ-VAS: EuroQol visual analogue scale (0–100).

### Psychological and sociodemographic profiles of PD and non-PD cases

Table 3 presents the differences between PD cases and non-PD cases in sociodemographic, mental and physical status, and emotion regulation and related factors. PD cases had significantly higher proportions of individuals who received higher education (47.3% vs 30.7%, *χ*^2^(1) = 6.24, *p* = 0.012) and had higher perceived socioeconomic status (7.0 vs 5.6, *t* = 7.24, *df* = 70.7, *p* < 0.001) than the non-PD cases. Compared to the non-PD class, the PD cases had less severe suicidal thoughts (8.1 vs 19.9, *t* = −10.31, *df* = 86.6, *p* < 0.001), less depression (10.0 vs 17.6, *t* = −9.65, *df* = 555, *p* < 0.001) and anxiety (7.6 vs 12.4, *t* = −6.52, *df* = 555, *p* < 0.001), more positive (15.3 vs 12.7, *t* = 5.91, *df* = 555, *p* < 0.001) and less negative affect (13.1 vs 16.5, *t* = −7.52, *df* = 555, *p* < 0.001).

**Table 3:** Characteristics of the participants based on class (n = 557).

	**PD cases (n = 55)**	**Non-PD cases (n = 502)**		
	**n (%)/M (SD)**	**n (%)/M (SD)**	**χ2**	***p***
Sex			0.12	.726
Male	10 (18.2%)	82 (16.3%)		
Female	45 (81.8%)	420 (83.7%)		
LGBTI status			2.13	.144
Yes	23 (41.8%)	262 (52.2%)		
No	32 (58.2%)	240 (47.8%)		
Location			3.00	.083
Rural/remote	7 (12.7%)	115 (22.9%)		
Metropolitan	48 (87.3%)	387 (77.1%)		
Living situation			0.05	.826
With family	40 (72.7%)	358 (71.3%)		
Others	15 (27.3%)	144 (28.7%)		
Relationship status			1.39	.238
Married, de facto, or in a relationship	27 (49.1%)	205 (40.8%)		
Others	28 (50.9%)	297 (59.2%)		
Education			**6.24**	**.012**
Diploma, bachelor, or above	**26 (47.3%)**	**154 (30.7%)**		
Others	**29 (52.7%)**	**348 (69.3%)**		
Physical health condition			1.11	.292
At least one long-term condition	16 (29.1%)	182 (36.3%)		
No long-term condition	39 (70.9%)	320 (63.7%)		
Mental health condition			3.67	.055
At least one diagnosis	36 (65.5%)	387 (77.1%)		
No diagnosed condition	19 (34.5%)	115 (22.9%)		
Current medication status			3.57	.059
Taking medication	21 (38.2%)	259 (51.6%)		
Not taking medication	34 (61.8%)	243 (48.4%)		
Age	22.2 (2.6)	21.9 (2.3)	0.99	.320
Socioeconomic status	**7.0 (1.4)**	**5.6 (1.6)**	**7.24**	** < .001**
SIDAS suicidal thoughts	**8.1 (7.5)**	**19.9 (11.8)**	**−10.31**	** < .001**
PHQ-9 depression	**10.0 (5.1)**	**17.6 (5.6)**	**−9.65**	** < .001**
GAD-7 anxiety	**7.6 (4.8)**	**12.4 (5.2)**	**−6.52**	** < .001**
PANAS-SF affect				
Positive affect	**15.3 (3.4)**	**12.7 (3.1)**	**5.91**	** < .001**
Negative affect	**13.1 (2.8)**	**16.5 (3.2)**	**−7.52**	** < .001**
DERS-16 emotion regulation	**46.8 (12.1)**	**57.6 (12.4)**	**−6.14**	** < .001**
CFS coping flexibility	**24.9 (5.1)**	**22.6 (4.6)**	**3.56**	** < .001**
CFS cognitive flexibility	**54.4 (6.8)**	**46.8 (7.4)**	**7.26**	** < .001**
RESE regulatory emotional self-efficacy				
Expressing positive affect	**14.6 (4.0)**	**11.2 (4.0)**	**6.00**	** < .001**
Managing despondency distress	**9.4 (3.3)**	**7.8 (3.3)**	**3.51**	** < .001**
Managing anger irritation	**10.3 (3.6)**	**8.8 (3.4)**	**3.08**	**.002**
Cognitive and behavioral responses				
Digital technology	**2.9 (0.7)**	**3.1 (0.6)**	**−2.38**	**.018**
Creative arts	2.2 (1.0)	2.1 (1.0)	0.39	.694
Self-harm and substance use	**1.9 (0.6)**	**2.5 (0.7)**	**−6.31**	** < .001**
Exercise	**2.1 (0.9)**	**1.8 (0.8)**	**3.36**	**.001**
Self-transcendence	**2.2 (0.6)**	**2.0 (0.5)**	**2.83**	**.005**
Self-care	2.6 (0.6)	2.6 (0.6)	0.74	.463

M: Mean; SD: standardized deviance; PD: positive deviant; LGBTI: lesbian, gay, bisexual, trans, and/or intersex; SIDAS: Suicidal Ideation Attributes Scale; PHQ-9: The Patient Health Questionnaire-9; GAD-7: General Anxiety Disorder-7; PANAS-SF: Positive and Negative Affect Schedule-Short Form; DERS-16: The Difficulties in Emotion Regulation Scale-16; CFS: Coping Flexibility Scale; CFS: Cognitive Flexibility Scale; RESE: Regulatory Emotional Self-efficacy Scale; Bold values indicate *p* < 0.05 based on independent groups t-tests for continuous variables and χ2 tests for categorical variables, comparing PD and non-PD group.

The PD cases reported lower levels of difficulties in emotion regulation (46.8 vs 57.6, *t* = −6.14, *df* = 555, *p* < 0.001), higher coping flexibility (24.9 vs 22.6, *t* = 3.56, *df* = 555, *p* < 0.001) and higher cognitive flexibility (54.4 vs 46.8, *t* = 7.26, *df* = 555, *p* < 0.001) than the non-PD cases. The PD cases also had higher levels of self-efficacy in expressing positive emotions (14.6 vs 11.2, *t* = 6.00, *df* = 555, *p* < 0.001), in managing despondency and distress (9.4 vs 7.8, *t* = 3.51, *df* = 555, *p* < 0.001), and in managing anger and irritation (10.3 vs 8.8, *t* = 3.08, *df* = 555, *p* = 0.002) than the non-PD cases. The PD cases were less likely to use digital technology (2.9 vs 3.1, *t* = −2.38, *df* = 555, *p* = 0.018) and less likely to use maladaptive coping behavior including self-harm and substance use (1.9 vs 2.5, *t* = −6.31, *df* = 555, *p* < 0.001) as a response to emotional distress. They were more likely to engage in physical exercise (2.1 vs 1.8, *t* = 3.36, *df* = 555, *p* = 0.001) and self-transcendence (2.2 vs 2.0, *t* = 2.83, *df* = 555, *p* = 0.005) than the non-PD cases. There was no significant difference between the PD and the non-PD cases on age, sex, sexual orientation, geographical location, living situation, relationship status, current medication status and the likelihood of engaging in creative arts or self-care activities under emotional distress.

### Predictors of the PD class

The predictors of the PD latent class are presented in Table 4. Having lower levels of emotion dysregulation, higher coping or cognitive flexibility, great self-efficacy in expressing positive affect, reduced use of digital technology, less self-harm or substance use as a response to emotional distress, more use of exercise and self-transcendence were associated with membership in the PD class. After adjustment for confounders (i.e., significant sociodemographic and mental health factors differentiating the PD and the non-PD classes), having higher cognitive flexibility, higher self-efficacy in expressing positive affect, reduced use of digital technology, and less self-harm and substance use were significantly associated with PD class membership.

**Table 4:** Logistic regression models of cognitive emotion regulation factors in predicting the PD latent class (n = 557).

	Unadjusted					Adjusted	
	OR	95% CI	*p*	OR		95% CI	*p*
DERS-16 emotion regulation	**0.937**	**0.916–0.958**	** < .001**	0.996		0.967–1.026	.783
CFS coping flexibility	**1.107**	**1.045–1.173**	**.001**	1.038		0.966–1.116	.304
CFS cognitive flexibility	**1.164**	**1.112–1.219**	** < .001**	**1.114**		**1.052–1.179**	** < .001**
RESE regulatory emotional self-efficacy							
Expressing positive affect	**1.219**	**1.127–1.318**	** < .001**	**1.119**		**1.024–1.222**	**.013**
Managing despondency distress	1.042	0.934–1.162	.464	0.944		0.831–1.073	.378
Managing anger irritation	1.064	0.954–1.187	.267	1.047		0.922–1.189	.475
Cognitive and behavioral responses							
Digital technology	**0.604**	**0.397–0.919**	**.019**	**0.474**		**0.282–0.798**	**.005**
Creative arts	1.057	0.803–1.391	.693	0.959		0.702–1.312	.796
Self-harm and substance use	**0.234**	**0.143–0.383**	** < .001**	**0.512**		**0.284–0.923**	**.026**
Exercise	**1.740**	**1.248–2.426**	**.001**	1.447		0.969–2.160	.071
Self-transcendence	**2.010**	**1.230–3.285**	**.005**	1.220		0.671–2.218	.514
Self-care	1.197	0.741–1.932	.462	0.934		0.543–1.606	.806

Bold values indicate *p* < .05; PD: Positive Deviant; DERS-16: The Difficulties in Emotion Regulation Scale-16; CFS: Coping Flexibility Scale; CFS: Cognitive Flexibility Scale; RESE: Regulatory Emotional Self-efficacy Scale; the results were adjusted for age, sex, education, social economic status, severity of suicidal thoughts, depression, and anxiety, and positive and negative affect.

## Discussion

This study investigated suicide resilience using a positive deviance (PD) informed analytic approach. Using latent class analysis, a single PD class, who had high levels of wellbeing and an absence of suicide attempts, was identified. The PD class constitutes 10% of the total sample. The PD class was found to have less severe suicidal thoughts, greater positive affect, and less negative affect than the non-PD class as expected. Few sociodemographic factors differentiated the PD group from the non-PD group, other than significantly greater rates of tertiary educational attainment and higher socioeconomic status.

Greater cognitive flexibility, greater self-efficacy in expressing positive affect, and adaptive emotion regulation strategies, including reduced use of digital technology and reduced self-harm and substance use, were significantly associated with membership of the PD class after adjusting for sociodemographic and mental health factors. The associations between PD class and emotion dysregulation, coping flexibility, increased use of exercise and self-transcendence were significant in bivariate analysis, but not significant in adjusted models. These findings indicate a number of potentially important foci for interventions to support individuals with current suicidal experiences.

Cognitive flexibility but not coping flexibility appears to be strongly associated with positive deviance. Approximately one standard deviation increase in cognitive flexibility was associated with double odds of positive deviance. This finding is in large consistent with the literature indicating that psychotherapies (e.g., cognitive behavioral therapy) that appear to increase cognitive flexibility^68^ are effective for reducing suicidal thoughts and behavior^69,70^. Although cognitive reframing is a key focus of CBT, the current findings suggest that providing an explicit focus on cognitive flexibility for people with suicidal thoughts may increase their resilience to suicidal behavior. Cognitive flexibility may be a potential mechanism as to how cognitive restructuring impacts suicidal thoughts and behavior but this explanation warrants further experimental investigation. We found no significant relationship between coping flexibility and positive deviance after adjusting for current mental health status and sociodemographic characteristics. This finding is consistent with previous findings^48^ indicating the moderating effects of coping flexibility on the relationship between depressive symptoms and suicide risks. Future studies may investigate the interaction between coping flexibility and mental health symptoms in individuals with suicidal thoughts or behavior in a larger sample size.

Our study indicates a significant relationship between regulatory emotional self-efficacy and PD membership, specifically through increased expression of positive affect. This finding echoes the increasing literature suggesting the important role of regulatory emotional self-efficacy in suicide prevention^71,72^. A recent study indicates significant mediating effects of regulatory emotional self-efficacy, but not acquired capability for suicide, on the relationships between nonsuicidal self-injury frequency and lifetime suicide attempts in both community-based and clinic samples^50^. Our findings extend this knowledge and suggest that regulatory emotional self-efficacy in expressing positive affect, but not self-efficacy in managing despondency distress or self-efficacy in managing anger irritation, may play a critical role in protecting young people from attempting suicide. Further studies to unveil the factors that differentiate the impacts of domains of regulatory emotional self-efficacy, for example, social support, self-esteem, or personality, may help in understanding the mechanisms underlying this association.

The findings surrounding the use of specific coping behavior also point to the focus areas where health promotion and clinical interventions may benefit young people at risk of suicide. Young adults in the PD class were less likely to use digital technology to cope with emotional distress. Although recent reviews indicate that the minimum impact of digital technology use on mental health symptoms^73,74^, our findings suggest that using digital technology (e.g., TV, online videos, social media) as a distraction to emotional distress can be a maladaptive coping strategy among young adults at risk of suicide. This is consistent with previous research reporting that the use of digital technology is not effective for coping with distress^75^ and is associated with lower levels of psychological wellbeing^76^.

Those in the PD class were also less likely to use substances or self-harm to cope with emotional distress, echoing previous findings on the roles of self-harm^77^ and substance use^78^ in suicidal behavior and a recent cohort study focusing on the transition from suicidal thoughts to behavior among adolescents^79^. Although physical activity and practice of self-transcendence (e.g., prayer, mindfulness or meditation) were significantly associated with the PD class in the bivariate associations, they became non-significant in the adjusted models. This finding is in large consistent with the qualitative research in suicide prevention^80^, suggesting the difficulties in practicing mindfulness and exercise during a crisis, particularly when fatigue is present. Nevertheless, physical activity has also been shown to improve cognitive flexibility in adults^68^, suggesting that behavioral activation and other health behavioral change approaches to increase physical activity may also aid in individuals’ coping with suicidal experiences, outside of a crisis.

Interestingly, emotion dysregulation was not significant in the adjusted model. Previous reviews have suggested that dialectical behavior therapy (DBT), which focuses on emotion regulation, is associated with only modest reductions in suicidal thoughts^70^, although the effects tend to be larger among people with personality disorders^81^. Our findings are consistent with previous findings that indicate: (1) that the relationship between emotion regulation and wellbeing is complex and is largely explained by mental health status^82^, (2) that emotion regulation may only impact wellbeing for certain subgroups of the population^83^, and (3) that observed effects are only related to specific emotion regulation strategies^83^. In combination, the findings suggest that a sole focus on emotion regulation is unlikely to be an optimal universal approach for suicide prevention interventions.

This study was the first to apply a positive deviance framework to identifying resilience to suicidal behavior, using a large nonclinical sample of young adults with recent suicidal thoughts. We found that 10% of the surveyed population met the criteria of PD. This number aligns with the previous estimate that PDs typically account for 0% to 10% of a population^84^. There were a few meaningful differences between the PD and the non-PD classes, which provides a potentially practical approach to revisiting the evidence in suicide prevention research. Whilst 90% of participants were identified as non-PD cases, it is important to note that 47% of them did not attempt suicide. Individuals who attempted suicide previously may also recover and never attempt suicide afterwards. Further investigation of the factors associated with suicide attempts in the non-PD subgroup may also be helpful for tailoring interventions.

Some limitations to this study need to be acknowledged. The direction and causation of significant associations could not be established due to the cross-sectional nature of the study. Nevertheless, the findings of this research suggest potential novel targets for further clinical, experimental and health promotion research. Examining longitudinal outcomes for people identified as positive deviants, including suicidal thoughts, suicide attempts and wellbeing, would also be an important extension of this research. Due to the epidemiological approach and distal assessment methodology adopted in this research, all the outcomes were based on self-report, without clinicians’ verification of mental health outcomes. Although established and validated measures were used, the examination of positive deviance in a clinical context may reveal other factors that support resilience to suicidal behavior. There may have been factors associated with positive deviance that we did not measure, which may be worthy of future investigation. Such factors may include personality, social connectedness, and hopelessness. Finally, it is unlikely that the recruitment strategy will result in a sample that was representative of the population of interest. We chose online recruitment because young adults have high use of social media and low rates of service use. Nevertheless, the variability in the use of social media and trust in advertising may have influenced the composition of the sample.

## Conclusions

This study demonstrated a methodology to understand suicidal resilience by identifying positive deviants, who comprised approximately 10% of a non-clinical sample with suicidal thoughts. We found that greater cognitive flexibility, greater self-efficacy in expressions of positive affect, reduced use of digital technology, and less self-harm and substance use for coping were associated with greater resilience to suicidal behavior. These findings suggest that specific emphasis on cognitive flexibility, regulatory emotional self-efficacy, and avoidance of maladaptive coping in therapeutic and health promotion interventions may be important for increasing wellbeing and reducing suicidal behavior among young adults. A greater focus on strengths-based approaches to suicide prevention may benefit young adults at risk of suicide.

## Data Availability

The data that support the findings of this study are available on request from the corresponding author, JH. The data are not publicly available due to ethics restrictions on the privacy of research participants.
